# Oleoylethanolamide Protects Against Acute Liver Injury by Regulating Nrf-2/HO-1 and NLRP3 Pathways in Mice

**DOI:** 10.3389/fphar.2020.605065

**Published:** 2021-01-18

**Authors:** Jiaji Hu, Zhoujie Zhu, Hanglu Ying, Jie Yao, Huabin Ma, Long Li, Yufen Zhao

**Affiliations:** Institute of Drug Discovery Technology, Ningbo University, Ningbo, China

**Keywords:** liver injury, oleoylethanolamide, oxidative stress, NLRP3 inflammasome, inflammation

## Abstract

Acute liver injury is a rapidly deteriorating clinical condition with markedly high morbidity and mortality. Oleoylethanolamide (OEA) is an endogenous lipid messenger with multiple bioactivities, and has therapeutic effects on various liver diseases. However, effects of OEA on acute liver injury remains unknown. In this study, effects and mechanisms of OEA in lipopolysaccharide (LPS)/d-galactosamine (D-Gal)-induced acute liver injury in mice were investigated. We found that OEA treatment significantly attenuated LPS/D-Gal-induced hepatocytes damage, reduced liver index (liver weight/body weight), decreased plasma alanine aminotransferase (ALT), aspartate aminotransferase (AST) and lactate dehydrogenase (LDH) levels. Moreover, mechanism study suggested that OEA pretreatment significantly reduced hepatic MDA levels, increased Superoxide dismutase (SOD) and Glutathione peroxidase (GSH-PX) activities via up-regulate Nrf-2 and HO-1 expression to exert anti-oxidation activity. Additionally, OEA markedly reduced the expression levels of Bax, Bcl-2 and cleaved caspase-3 to suppress hepatocyte apoptosis. Meanwhile, OEA remarkedly reduced the number of activated intrahepatic macrophages, and alleviated the mRNA expression of pro-inflammatory factors, including TNF-α, IL-6, MCP1 and RANTES. Furthermore, OEA obviously reduced the expression of IL-1β in liver and plasma through inhibit protein levels of NLRP3 and caspase-1, which indicated that OEA could suppress NLRP3 inflammasome pathway. We further determined the protein expression of PPAR-α in liver and found that OEA significantly increase hepatic PPAR-α expression. In addition, HO-1 inhibitor ZnPP blocked the therapeutic effects of OEA on LPS/D-Gal-induced liver damage and oxidative stress, suggesting crucial role of Nrf-2/HO-1 pathway in the protective effects of OEA in acute liver injury. Together, these findings demonstrated that OEA protect against the LPS/D-Gal-induced acute liver injury in mice through the inhibition of apoptosis, oxidative stress and inflammation, and its mechanisms might be associated with the Nrf-2/HO-1 and NLRP3 inflammasome signaling pathways.

## Introduction

Acute liver injury, also named acute liver failure, is a dramatic clinical syndrome associated with severe liver dysfunction and high mortality rates generally resulting from sepsis, viral hepatitis, autoimmune hepatitis, alcohol abuse or drugs overdose (Bernal and Wendon, 2013; Stravitz and Lee, 2019). Unfortunately, effective pharmacological strategies for clinical treatment of acute liver injury are still urgently needed (Dong et al., 2020). Lipopolysaccharide (LPS), an endotoxin component of Gram-negative bacterial cell wall, can induce acute liver injury in mice together with d-Galactosamine (D-Gal). This widely used animal model could accurately mimic all the complications of the fulminant liver failure in human, including hepatocyte apoptosis, intracellular oxidative stress and inflammation (Lei et al., 2020; Yang et al., 2020).

Oxidative stress as a pivotal factor in the development of acute liver injury has been widely described (Ramachandran and Jaeschke, 2018). Therapeutic strategies that attenuate oxidative stress are important in managing liver injury (Li et al., 2015b; Zhao et al., 2019). Previous studies have reported that the redox-responsive transcription factor Nuclear factor-E2-related factor 2 (Nrf-2) and the anti-oxidant enzyme Heme Oxygenase-1(HO-1) play a crucial role in the regulation of oxidative stress during the pathogenesis of liver injury (Ge et al., 2017; Saeedi et al., 2020). When activated by the oxidative stress, Nrf-2 can transfer from the cytoplasm into the nucleus, and then binding with the antioxidant responsive elements (AREs), leading to the transcription of HO-1 and some other anti-oxidative genes (Loboda et al., 2016; Liu et al., 2019). In addition to HO-1, the superoxide dismutase (SOD) and glutathione peroxidase (GSH-px) also play an important role in the protection of cells against oxidative damage as anti-oxidative enzymes (Olsvik et al., 2005; Damiano et al., 2018). Therefore, the elevated levels of SOD and GSH-px generally reflect enhanced abilities against oxidative stress.

The NACHT, LRR, and PYD domains-containing protein 3 (NLRP3) inflammasome is a kind of cytoplasmic multiprotein complex, which could be activated under pathogen-associated molecular patterns (PAMPs) or damage-associated molecular patterns (DAMPs) (Li et al., 2020b). Upon activation, the NLRP3 inflammasome recruit the effector molecular caspase-1through the adaptor molecule apoptosis-associated speck-like protein containing a CARD (ASC), and then promote the expression and secretion of pro-inflammatory cytokines, including interleukin-18 (IL-18) and interleukin-1β (IL-1β), which play key effects in the pathogenesis of many inflammation-associated diseases (He et al., 2020). Many previous studies have demonstrated the key role of NLRP3 inflammasome in the progress of all kinds of liver disease models including LPS/D-Gal-induced acute liver injury (Kim and Lee, 2013; Liu et al., 2018; Jimenez Calvente et al., 2020). Therefore, inhibiting the activation of NLRP3 inflammasome could be an effective strategy for the treatment of acute liver injury.

Oleoylethanolamide (OEA), an endogenous bioactive lipid molecular binds with high affinity to the nuclear receptor peroxisome proliferator-activated receptor-α (PPAR-α), exhibits plentiful pharmacological activities, including anti-obesity, anti-inflammation and anti-oxidant effects. An increasing number of studies have demonstrated the therapeutic effects of OEA in the modulating of multiple liver diseases. For instance, Pan et al. have reported that OEA reduces lipid synthesis and lipoprotein secretion in hepatocytes (Pan et al., 2018). A recent study indicated that OEA promotes fasting-induced liver ketogenesis through activate PPAR-α(Misto et al., 2019). Our previous studies have demonstrated that OEA could improve high fat diet-induced liver steatosis in rats, and also can significantly alleviate methionine choline-deficient diet-induced and thioacetamide-induced liver fibrosis through PPAR-α mediated inhibition of hepatic stellate cells activation (Li et al., 2015a; Chen et al., 2015). However, the effects of OEA in acute liver injury still not be well described. In the present study, we explored the potential beneficial therapeutic effects of OEA against LPS/D-Gal-induced acute liver injury. In addition, we demonstrated the molecular mechanisms underlying these effects that OEA could significantly suppress hepatocyte apoptosis, reduce oxidative stress, inhibit inflammation and markedly attenuate NLRP3 inflammasome activation. Moreover, OEA can obviously promote PPAR-α expression in the injured liver.

## Materials and Methods

### Animal Experiments

The adult C57BL/6 mice at age of 6–8 weeks were purchased from Beijing Vital River Laboratory Animal Technology Co., Ltd. (Beijing, China). All experiments for animal care and use were conducted in accordance with the approved guidelines by the Committee for Animal Research at Ningbo University. The mice were given water ad libitum, housed and kept in an environment with constant temperature (21–23 °C) and humidity (55–60%) under 12 h light/dark cycles. Acute liver injury model was induced in mice by intraperitoneal injection with LPS (50 μg/kg body weight) and D-Gal (400 mg/kg body weight) which were both dissolved in normal saline. OEA was dissolved in the vehicle containing 10% Tween-80 + 10% PEG-400 + 80% saline at 1 mg/ml. For the OEA pretreatment experiment, OEA (10 mg/kg body weight) was given to the mice at 2 h before LPS/D-Gal injection with a volume of 10 ml/kg, and the mice were sacrificed 5 h after LPS/D-Gal administration. Mice were each randomly divided into three groups (*n* = 6–8/group): 1) control group only received saline; 2) Model group received LPS/D-Gal and the vehicle of OEA; 3) OEA group received OEA treatment and LPS/D-Gal injection. For the OEA therapeutic experiment, mice were randomly divided into five groups: 1) control group; 2) OEA control group; 3) Model group; 4) OEA + LPS/D-Gal group; 5) OEA + LPS/D-Gal + ZnPP group. ZnPP was first dissolved in 0.2M NaOH, then adjusted the Ph to 7.4 with HCL, and diluted with saline to the concentration of 1 mg/ml. Mice were pretreated with ZnPP (10 mg/kg body weight) or the vehicle of ZnPP at 30 min before LPS/D-Gal injection, OEA or the vehicle of OEA was intraperitoneally administered immediately after LPS/D-Gal treatment, the animals were sacrificed 5 h after OEA treatment.

### Chemicals and Reagents

OEA synthesis was carried out as previously described (Li et al., 2015a). Lipopolysaccharide (LPS), d-galactosamine (D-Gal), Zinc protoporphyrin IX (ZnPP) and all other chemicals were acquired from Sigma–Aldrich (Shanghai, China). Malondialdehyde (MDA), Superoxide dismutase (SOD), Glutathione peroxidase (GSH-PX), Alanine transaminase (ALT), Aspartate transaminase (AST), and lactate dehydrogenase (LDH) assay kits were obtained from Nanjing Jiancheng Bioengineering Institute (Nanjing, China) and Beijing Solarbio Science & Technology Co., Ltd. (Beijing, China). Cell Counting Kit-8 (CCK-8) was obtained from Beyotime Institute of Biotechnology (Shanghai, China). Anti-Cleaved caspase3 antibody (clone 5A1E) was purchased from Cell Signaling Technology (Shanghai, China). Anti-NLRP3 antibody (#A5652) was purchased from Abclonal (Wuhan, China). Anti-Caspase1 antibody (clone 14F468) was purchased from Santa cruz (Shanghai, China). Anti-PPAR-α antibody (#ab24509) was purchased from Abcam (Shanghai, China). Antibodies against Bax (#50599-2-Ig), Bcl-2 (#12789-1-AP), Nrf-2 (#16396-1-AP), HO-1 (#10701-1-AP), β-actin (clone 2D4H5) and GAPDH (clone 1E6D9) were purchased from Proteintech (Wuhan, China). HRP-conjugated goat anti-rabbit and goat anti-mouse antibodies were obtained from Proteintech (Wuhan, China).

### TUNEL Assay

Apoptosis in liver was examined using terminal deoxynucleotidyl transferase-mediated dUTP nick end labeling (TUNEL) assay kit (Roche, Shanghai, China) as manufacturer’s protocol. The sections were counterstained with 4′-6-diamidino-2-phenylindole (DAPI) (Vector Lab, Shenzhen, China). The specimens were observed and imaged with the Axio Observer microscope with ApoTome (Carl Zeiss, Jena, Germany).

### Histological and Immunohistochemistry (IHC) Analysis

Liver lobe samples were collected at termination and fixed in the 4% paraformaldehyde for 24 h, embedded with paraffin, sectioned at 5 μm using Leica SM2010 R Sliding microtome (Shanghai, China), and stained with hematoxylin and eosin (HE) to assess histological features. The histological analysis of acute liver injury was scored according to Suzuki’s criteria, which evaluate the grades of liver damage from 0 to 4 through analyze the degree of hepatocyte necrosis, hemorrhage, and vacuolation as follows: 0, no injury; 1, minimal injury; 2, mild injury; 3, moderate injury; 4, severe injury. For immunohistochemistry staining, liver sections were blocked with goat serum after heating 10 min in sodium citrate buffer (pH = 6.0) for antigen retrieval, and incubated with anti-F4/80 (clone CI:A3-I, 1:200 dilution, Abcam, Shanghai, China) or anti-IL-1β (polyclone, 1:200 dilution, Abcam, Shanghai, China) antibodies at 4 °C overnight. The immunostained areas were determined using HRP/DAB Detection Kit (MXB Biotechnologies, Fuzhou, China) and counterstained with hematoxylin. The species-matched immunoglobulin G were used as negative control antibodies in each IHC assay to check the specificity of the primary antibodies on mouse liver sections. The images were captured under a standard microscope (Leica, Shanghai, China). To quantify F4/80 and IL-1β positive area in total area of liver sections, we randomly examined five fields per slide in five mice of each group using the software ImageJ.

### Biochemical Analysis and ELISA Assays

Plasma ALT, AST levels and hepatic activity levels of SOD, MDA, and GSH-PX were analyzed with colorimetric assay kits (Nanjing Jiancheng Bioengineering Institute, Nanjing, China) in accordance with the manufacturer’s guidelines. The protein levels of plasma IL-1β were analyzed using enzyme-linked immunosorbent assay (ELISA) kit (R&D Systems, Shanghai, China) according to the manufacturer’s protocol. All biochemistry and ELISA assays were determined by a SpectraMax Paradigm Multi-Mode Microplate Reader (Molecular Devices, Shanghai, China).

### Western Blot Analysis

Western blot analysis was conducted as previously stated (Li et al., 2018). Briefly, proteins were separated by SDS-PAGE and then transferred to PVDF membranes. These membranes were blocked by 5% BSA for 1 h followed with incubation of primary antibodies including anti-Cleaved caspase3 (1:1,000), anti-NLRP3 (1:1,000), anti-Caspase1 (1:500), anti-PPAR-α (1:1,000), anti-Bax (1:5,000), anti-Bcl-2 (1:1,000), anti-Nrf-2 (1:1,000), anti-HO-1 (1:1,000), anti-GCLC (1:1,000), β-actin (1:5,000) and GAPDH (1:10,000) overnight at 4°C. After washing 3 times, the membranes were incubated with HRP-conjugated secondary antibodies for 1 h. Finally, target proteins were obeserved by enhanced chemiluminescence (ECL) with ChemiDoc XRS system (Bio-Rad, Shanghai, China), all the protein expression levels were normalized to the expression level of GAPDH or β-actin.

### Real Time PCR

Total RNA samples from liver tissues or cells was extracted with the RNA simple Total RNA Kit (Tiangen, Beijing, China) and was synthesized to cDNA with a FastQuant RT kit (Tiangen, Beijing, China) following the manufacturer’s protocol. relative quantitation of mRNA was performed on a CFX Connect Real-Time PCR Detection System (Bio-Rad, Shanghai, China) using SuperReal PreMix Plus (SYBR Green) kit (Tiangen, Beijing, China) according to the manufacturer’s protocol. The levels of mRNA were normalized to GAPDH. The sequence of primers as follows: TNF-α: forward: CAG​GCG​GTG​CCT​ATG​TCT​C; reverse: CGA​TCA​CCC​CGA​AGT​TCA​GTA​G. IL-6: forward: AAT​TAA​GCC​TCC​GAC​TTG​TGA​AG; reverse: CTT​CCA​TCC​AGT​TGC​CTT​CTT​G. RANTES: forward: GCT​GCT​TTG​CCT​ACC​TCT​CC; reverse: TCG​AGT​GAC​AAA​CAC​GAC​TGC. MCP-1: forward: TTA​AAA​ACC​TGG​ATC​GGA​ACC​AA; reverse: GCA​TTA​GCT​TCA​GAT​TTA​CGG​GT. IL-1β: forward: GAA​ATG​CCA​CCT​TTT​GAC​AGT​G; reverse: TGG​ATG​CTC​TCA​TCA​GGA​CAG.

### Cell Culture and Treatment

Murine liver cell line alpha mouse liver 12 (AML12) and macrophage cell line RAW264.7 were obtained from American Type Culture Collection (Manassas, United States). AML12 cells were cultured in Dulbecco's modified Eagle medium (DMEM) and Ham’s F12 medium (1:1) with 10% fetal bovine serum (FBS), 0.05 mg/ml insulin, 0.005 mg/ml transferrin, 0.005 μg/ml selenium and 0.04 μg/ml dexamethasone. RAW264.7 cells were cultured in DMEM with 10% FBS. All the cells were maintained in an environment with 5% CO_2_ at 37 °C. For the experiment of H_2_O_2_-induced cell injury, the AML12 cells were treated with OEA (3, 10, 30, 100 μM) at 2 h before H_2_O_2_ (300 μM) stimulation, and further cultured for another 24 h, the cell viability was analyzed using CCK-8 kit. For the experiment of LPS-induced inflammatory response, the RAW264.7 macrophages were treated with LPS (100 ng/ml) for 24 h in the presence or absence of OEA, to analyze the effect of OEA on LPS-induced inflammatory factors expression.

### Statistical Analysis

All the data were expressed as the mean ± SEM. Statistical differences were evaluated using GraphPad Prism version 8.3.0 for Windows. One-way analysis of variance (ANOVA) followed by the Bonferroni multiple comparison test was used for data comparison. Statistically significant were defined as *p* value less than 0.05.

## Results

### OEA attenuated LPS/D-Gal-Induced Acute Liver Injury in Mice

**FIGURE 1:**
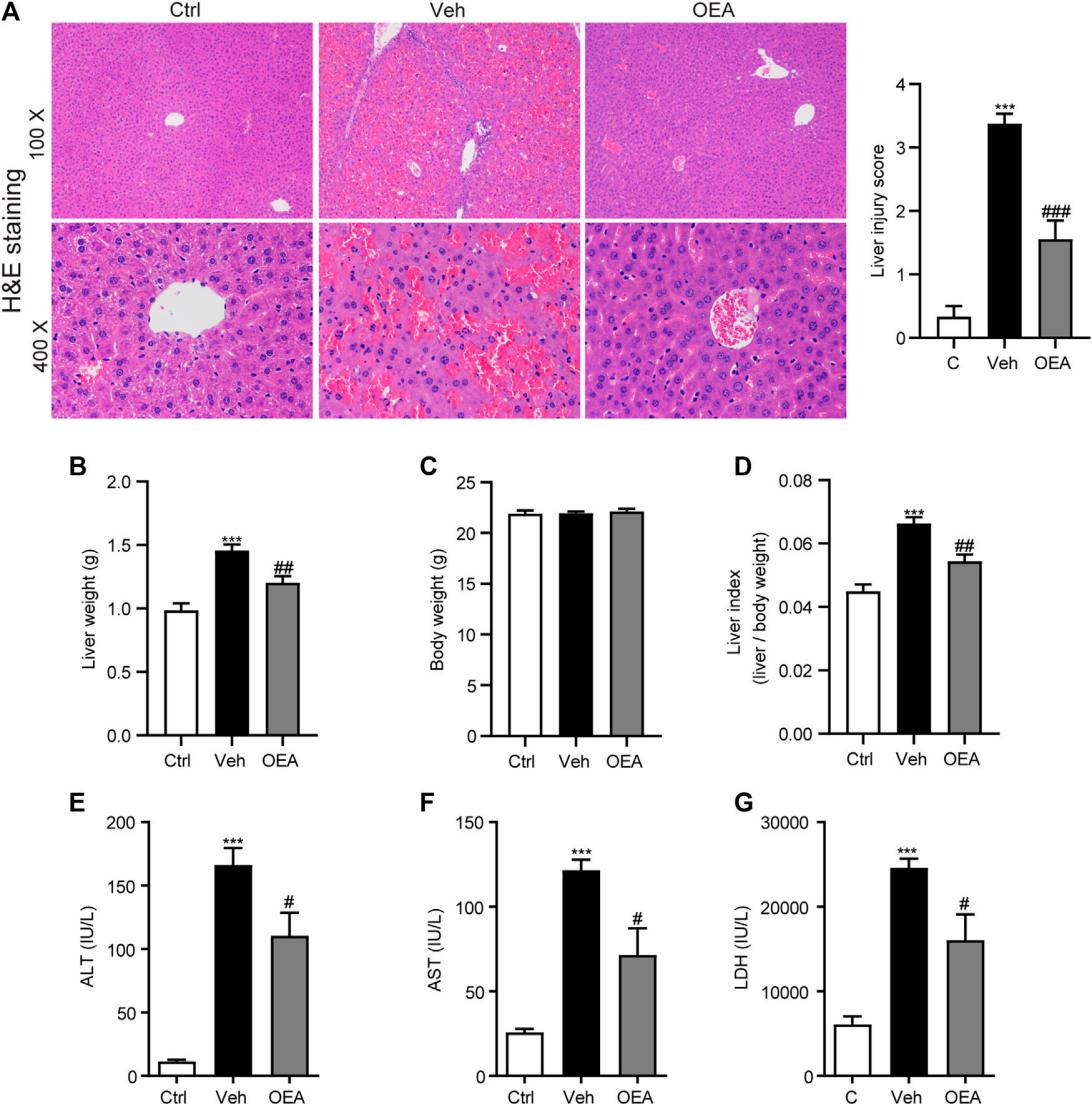
OEA inhibits LPS/D-Gal-induced acute liver injury in mice. **(A)** Hematoxylin-eosin (HE) staining (original magnifications, ×100 × 400) of liver tissues from mice of Ctrl, Veh and OEA group. **(B)** Liver weight. **(C)** Body weight. **(D)** Liver index (liver/body weight). **(E)** Plasma ALT levels. **(F)** Plasma AST levels. **(G)** Plasma LDH levels. Ctrl: control group; Veh: vehicle group treated with LPS/D-Gal and vehicle; OEA: OEA group treated with LPS/D-Gal and OEA. Data are expressed as mean ± SEM, *n* = 6 for Ctrl group, *n* = 8 for Veh and OEA group. ***p < 0.001, compared with the Ctrl group, #*p* < 0.05, ##*p* < 0.01, ###*p* < 0.001, compared with the Veh group.

To detect the protective role of OEA against acute liver injury induced by LPS/D-Gal injection, we examined histopathological changes, liver weight, body weight and calculated the liver index, the plasma ALT, AST, and LDH levels were also analyzed. As shown in Figure 1A, LPS/D-Gal stimulation significantly increased the liver injury score, as indicated by serious hepatic lobule disorder, obvious intrahepatic congestion, hemorrhage and edema. However, OEA treatment significantly decreased liver histopathological score. The results of liver weight, body weight and liver index showed that pretreatment of OEA markedly attenuated LPS/D-Gal-induced liver weight and liver index upregulation (Figures 1B–D). The plasma levels of ALT, AST, and LDH were obviously elevated post LPS/D-Gal administration. However, pretreatment of OEA dramatically alleviated the plasma ALT, AST, and LDH levels than in the LPS/D-Gal group. Those results above demonstrated that OEA exerts remarkable protective effects on acute liver injury in LPS/D-Gal-treated mice.

### OEA Ameliorated Liver Apoptosis in LPS/D-Gal Treated Mice

**FIGURE 2:**
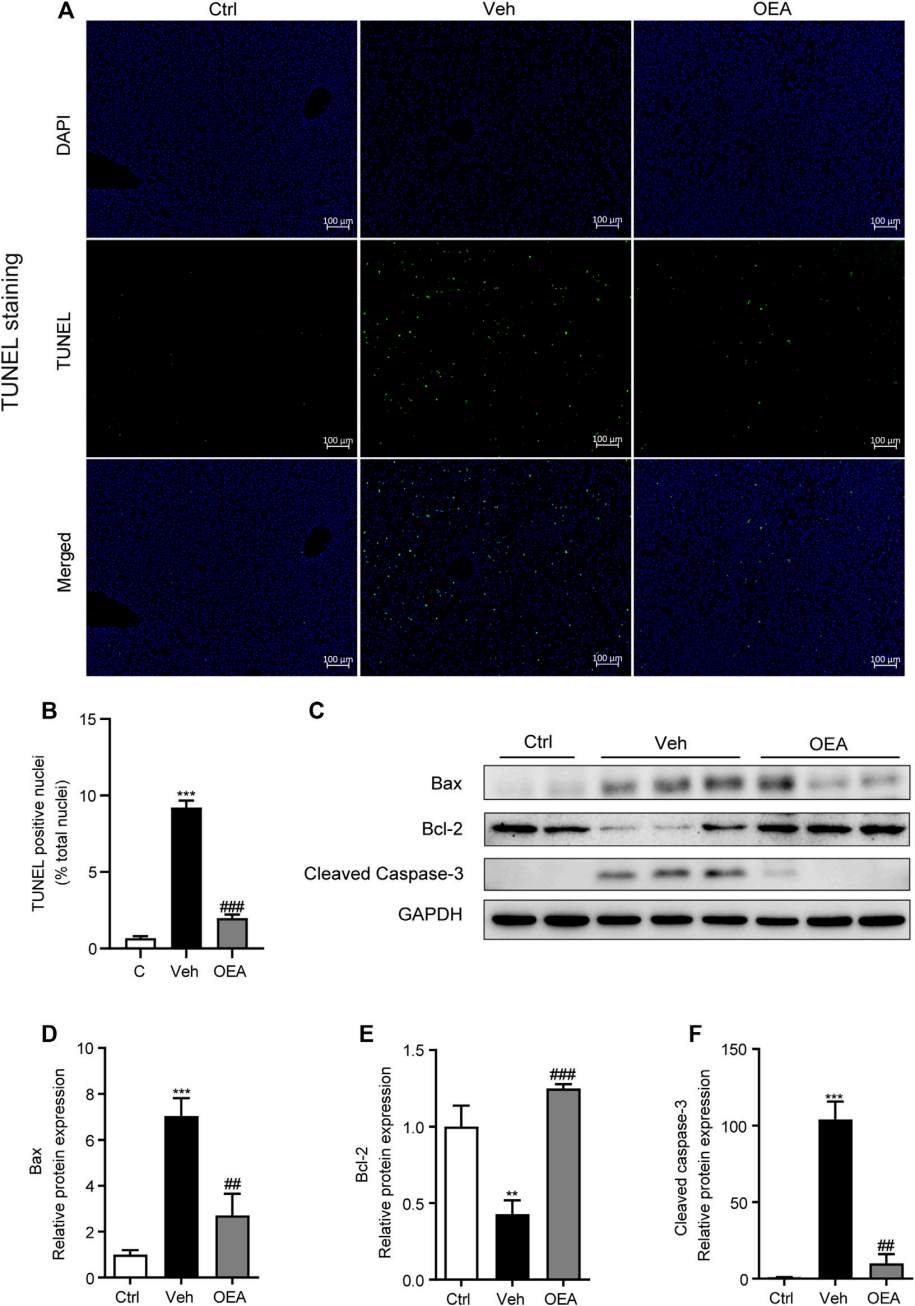
Effect of OEA on LPS/D-Gal-induced apoptosis in liver. **(A,B)** TUNEL staining (original magnifications, ×100) of liver tissues counterstained with DAPI. **(C–F)** Protein expression of hepatic Bax, Bcl-2 and Cleaved Caspase-3 were detected by western blot. Ctrl: control group; Veh: vehicle group treated with LPS/D-Gal and vehicle; OEA: OEA group treated with LPS/D-Gal and OEA. Data are expressed as mean ± SEM, *n* = 6 for Ctrl group, *n* = 8 for Veh and OEA group. **p < 0.01, ***p < 0.001, compared with the Ctrl group, ##*p* < 0.01, ###*p* < 0.001, compared with the Veh group.

Liver apoptosis plays a very important role in the pathological process of acute liver injury. To investigate the effects of OEA on LPS/D-Gal-induced liver apoptosis, we determined the proportion of apoptotic hepatocytes in liver using TUNEL staining analysis. Compared with the control group, LPS/D-Gal remarkably increased the numbers of TUNEL-positive cells in liver, whereas OEA pretreatment significantly reduced these numbers (Figures 2A,B, Supplemental Figure S1). Western blot analysis results showed that protein expression of Bax was significantly increased after LPS/D-Gal stimulation, while the protein expression of Bcl-2 markedly decreased in the vehicle group, which indicated the existence of apoptosis in accordance with previous reports (Song et al., 2019). However, OEA pretreatment significantly alleviated the protein expression of Bax and Bcl-2 in the liver (Figures 2C–E). Caspase-3 is a frequently activated cysteine–aspartic acid protease that related to apoptosis, while cleaved caspase-3 is the activated form of caspase-3 to cleave downstream targets and execute cell death (Wang, 2014). Here we found that hepatic protein levels of cleaved caspase-3 were obviously elevated after the stimulation of LPS/D-Gal, while OEA treatment significantly reduced this elevation (Figure 2F). These data indicated that OEA can ameliorate hepatic apoptosis during acute liver injury.

### OEA Alleviated LPS/D-Gal-Induced Oxidative Stress

**FIGURE 3:**
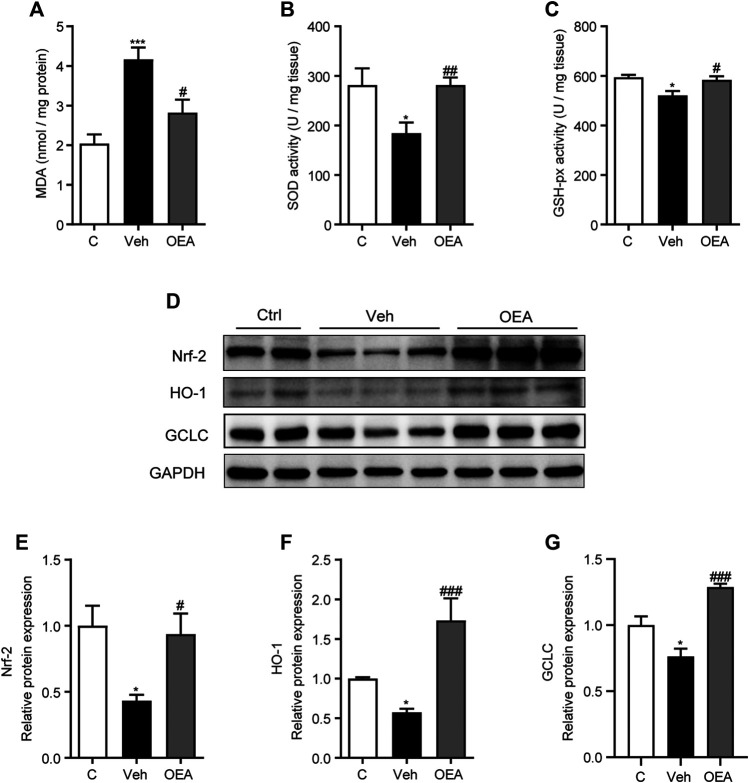
OEA regulates hepatic oxidative stress induced by LPS/D-Gal. **(A)** Hepatic MDA levels. **(B)** Hepatic SOD activity. **(C)** Hepatic GSH-px activity. **(D–G)** Nrf-2, HO-1 and GCLC protein levels in liver determined by western blot. Ctrl: control group; Veh: vehicle group treated with LPS/D-Gal and vehicle; OEA: OEA group treated with LPS/D-Gal and OEA. Data are expressed as mean ± SEM, *n* = 6 for Ctrl group, *n* = 8 for Veh and OEA group. *p < 0.05, ***p < 0.001, compared with the Ctrl group, #*p* < 0.05, ##*p* < 0.01, ###*p* < 0.001, compared with the Veh group.

Oxidative stress is established in either acute or chronic liver injury model both *in vitro* and *in vivo*, and play an important role during the occurrence and development of these pathological process. It was proved useful to attenuate acute liver injury with anti-oxidant system (Qi et al., 2019; Li et al., 2020c). To investigate the effects of OEA on oxidative stress in acute liver injury, we detected the hepatic MDA levels, SOD activities and GSH-hx activities at 5 h post LPS/D-Gal treatment. Hepatic MDA content is a most prevalent biomarker of lipid peroxidation during liver injury (Gawel et al., 2004; Kilany et al., 2020). As shown in Figure 3A, hepatic MDA levels significantly increased in the vehicle group than in the control group, and this increased expression was decreased by OEA pretreatment. SOD and GSH-px are the major anti-oxidant enzymes in the liver. In our study, LPS/D-Gal treatment resulted in a significant decrease of hepatic SOD and GSH-px activities, while OEA pretreatment markedly upregulated the activities of SOD and GSH-px (Figures 3B,C). Nrf-2/HO-1 signaling pathway is the most important pathway which involved in oxidative stress. We determined the protein expression levels of Nrf-2, HO-1 and GCLC, and found that these proteins were all markedly decreased in vehicle group received LPS/D-Gal injection. Additionally, OEA dramatically reactivated the expression of Nrf-2, HO-1 and GCLC in the liver (Figures 3D–G). These results suggested that OEA could attenuate hepatic oxidative stress through Nrf-2/HO-1 pathway in LPS/D-Gal-induced acute liver injury.

### OEA Inhibited LPS/D-Gal Induced Inflammation in Mice

**FIGURE 4:**
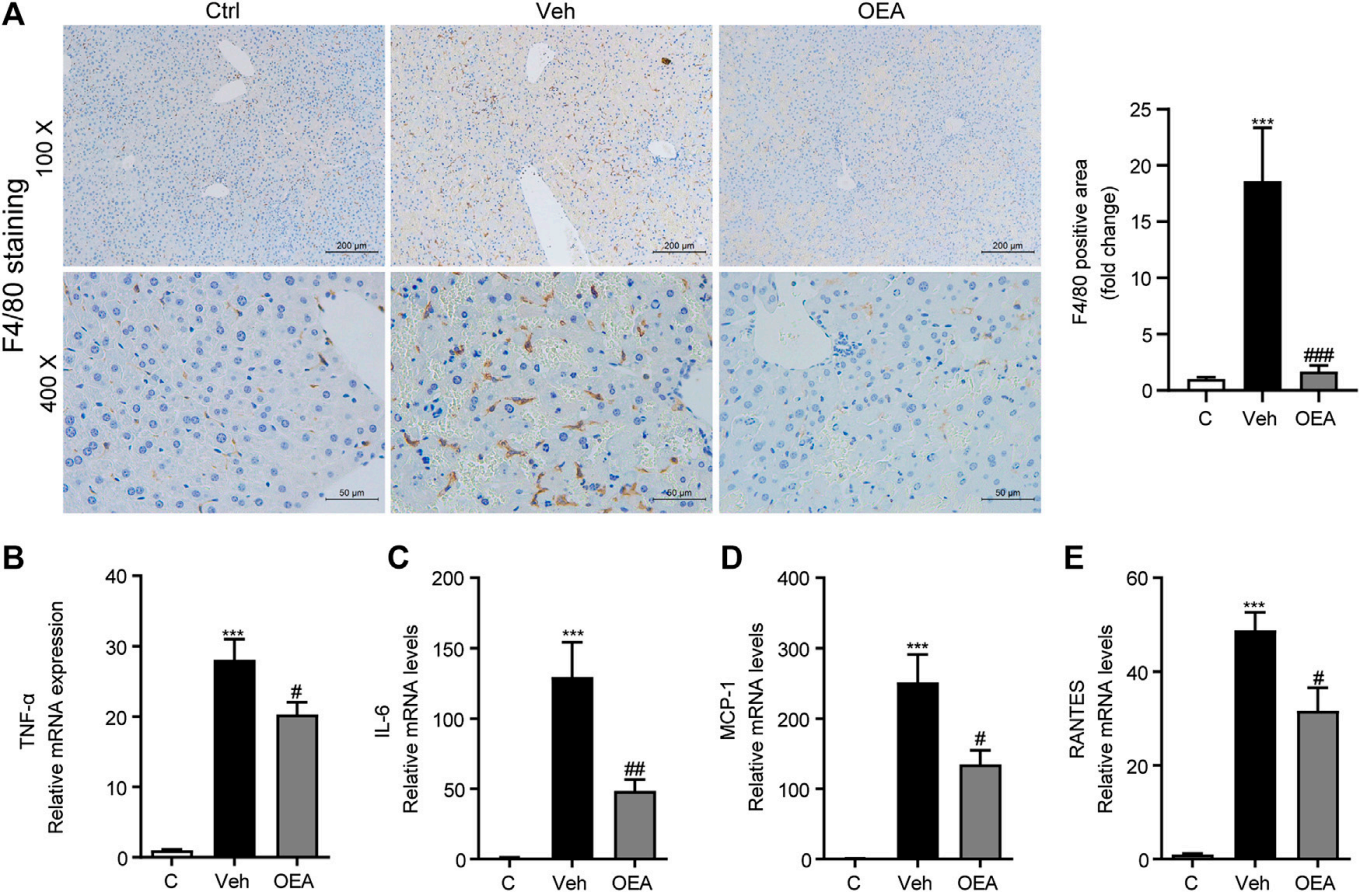
OEA ameliorates LPS/D-Gal induced-hepatic inflammatory responses. **(A)** Immunochemistry staining (original magnifications, ×100, ×400) of F4/80 in liver. **(B–E)** TNF-α, IL-6, MCP-1 and RANTES mRNA expression in liver detected by real time PCR. Ctrl: control group; Veh: vehicle group treated with LPS/D-Gal and vehicle; OEA: OEA group treated with LPS/D-Gal and OEA. Data are expressed as mean ± SEM, *n* = 6 for Ctrl group, *n* = 8 for Veh and OEA group. ***p < 0.001, compared with the Ctrl group, #*p* < 0.05, ##*p* < 0.01, ###*p* < 0.001, compared with the Veh group.

LPS is an important pathogenic stimulator that could obviously induce the activation of macrophage and induce the expression of pro-inflammatory factors in liver, and these inflammatory responses could aggravate the development of liver injury (Engelmann et al., 2020). F4/80 is the most classical biomarker in activated macrophage, to investigate whether OEA affect the activation of intrahepatic macrophages, here we first detected F4/80 expression in liver using IHC staining analysis, and found that there are much more F4/80-positive cells distributed in the liver after LPS/D-Gal stimulation, while OEA treatment significantly attenuated F4/80 expression in the liver (Figure 4A). In accordance with the increased number of activated macrophages, the LPS/D-Gal injection also dramatically up-regulated the expression of pro-inflammatory factors in liver, including TNF-α, IL-6, MCP1 and RANTES. As expected, OEA significantly reduced the expression of these inflammatory mediators (Figures 4B–E). The results above indicated that OEA could inhibit hepatic inflammatory response during acute liver injury in mice.

### OEA Attenuated LPS/D-Gal-Induced NLRP3 Inflammasome Activation

**FIGURE 5:**
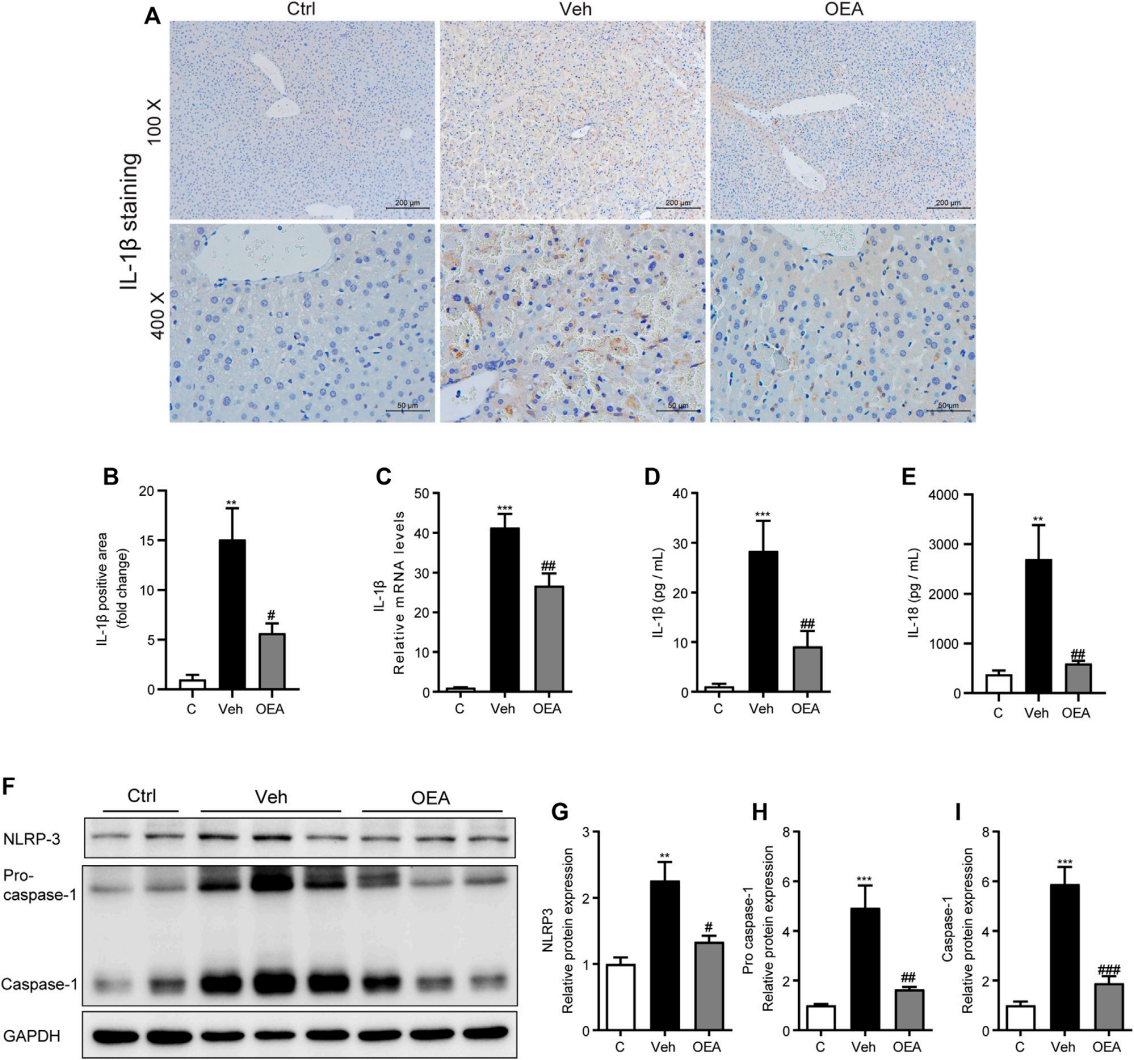
OEA attenuates LPS/D-Gal induced-NLRP3 inflammasome activation. **(A,B)** Immunochemistry staining (original magnifications, ×100, ×400) of IL-1β in liver. **(C)** IL-1β mRNA expression in liver. **(D)** Plasma IL-1β concentrations. **(E)** Plasma IL-18 concentrations. **(F–I)** Protein expression of NLRP3, Pro-caspase-1 and Caspase-1 levels in liver. Ctrl: control group; Veh: vehicle group treated with LPS/D-Gal and vehicle; OEA: OEA group treated with LPS/D-Gal and OEA. Data are expressed as mean ± SEM, *n* = 6 for Ctrl group, *n* = 8 for Veh and OEA group. **p < 0.01, ***p < 0.001, compared with the Ctrl group, #*p* < 0.05, ##*p* < 0.01, ###*p* < 0.001, compared with the Veh group.

Previous reports have showed that LPS/D-Gal-induced inflammatory response in the liver may due to NLRP3 inflammasome activation (Kim and Lee, 2013). To investigate the effect of OEA on the activation of NLRP3 inflammasome, we detected the protein and mRNA levels of IL-1β, which is an inflammatory cytokine mediated the development of liver injury. IHC staining revealed that protein expression of IL-1β in the liver was dramatically raised in the vehicle group compare to the control group, while significantly reduced in the OEA group compare to the control group (Figures 5A,B). It is showed that the mRNA levels of IL-1β in the liver were also markedly increased during the LPS/D-Gal injection, and decreased obviously after OEA treatment (Figure 5C). As a cytokine, IL-1β could be released to the circulatory system. To detect the levels of IL-1β distributed in the whole body, we analyzed the plasma IL-1β levels via ELISA assay. As shown in Figure 5D, plasma IL-1β levels were notably elevated in LPS/D-Gal treated mice, and these increases were inhibited by OEA pretreatment. The mice exposed to LPS/D-Gal also exhibited elevated levels of plasma IL-18, which were decreased in mice were given OEA pretreatment (Figure 5E). Consistent with the expression of IL-1β, the protein levels of NLRP3 inflammasome components NLRP3, Pro-caspase-1 and Caspase-1 were also markedly raised after LPS/D-Gal injection. The increase of these proteins was alleviated by OEA treatment (Figures 5F–I). These results suggested that OEA attenuated NLRP3 inflammasome activation in acute liver injury mice.

### OEA Restore PPAR-α Expression in Liver Tissue

**FIGURE 6:**
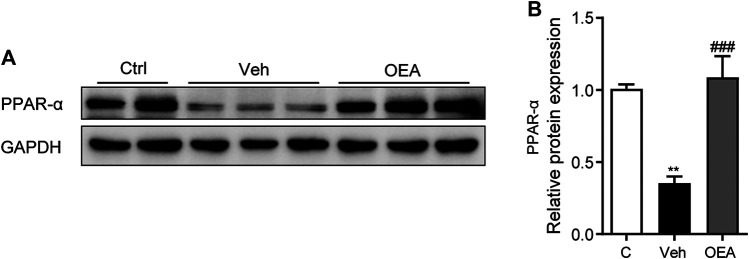
OEA enhance PPAR-α expression during acute liver injury. **(A,B)** Protein expression of hepatic PPAR-α levels in liver. Ctrl: control group; Veh: vehicle group treated with LPS/D-Gal and vehicle; OEA: OEA group treated with LPS/D-Gal and OEA. Data are expressed as mean ± SEM, *n* = 6 for Ctrl group, *n* = 8 for Veh and OEA group. **p < 0.01, compared with the Ctrl group, ###*p* < 0.001, compared with the Veh group.

To determine the mechanisms of whether PPAR-α participated in the effect of OEA on acute liver injury, we detected the protein expression of PPAR-α in the liver. Here we discovered that LPS/D-Gal treatment significantly suppressed hepatic PPAR-α expression, while OEA remarkedly increased the expression of PPAR-α (Figures 6A,B). These findings indicated that the protective effects of OEA on LPS/D-Gal induced acute liver injury may mediated by PPAR-α signaling pathway.

### HO-1 Pathway Mediated the Therapeutic Effects of OEA on LPS/D-Gal-Induced Liver Injury

**FIGURE 7:**
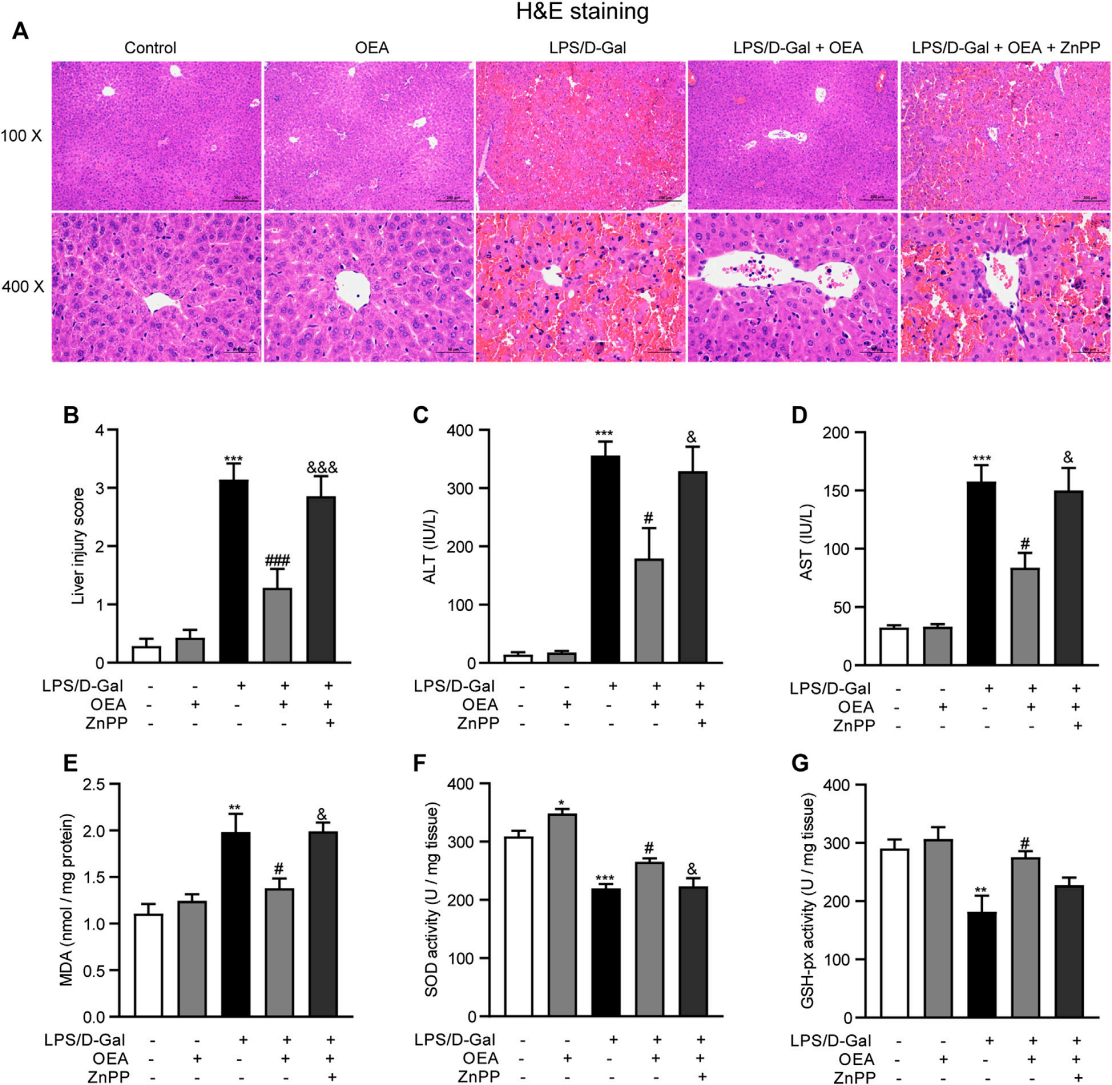
HO-1 inhibition blocked the protective effects of OEA in acute liver injury. **(A)** Hematoxylin-eosin (HE) staining (original magnifications, ×100 × 400) of liver tissues from mice of Control, OEA-Control, LPS/D-Gal, LPS/D-Gal + OEA, and LPS/D-Gal + OEA + ZnPP group. **(B)** Liver injury score. **(C)** Plasma ALT levels. **(D)** Plasma AST levels. **(E)** Hepatic MDA levels. **(F)** Hepatic SOD activity. **(G)** Hepatic GSH-px activity. Data are expressed as mean ± SEM, *n* = 6. *p < 0.05, **p < 0.01, ***p < 0.001, compared with the Control group, #*p* < 0.05, ###*p* < 0.001, compared with the LPS/D-Gal group, &*p* < 0.05, &&&*p* < 0.001, compared with the LPS/D-Gal + OEA group.

To investigate whether OEA has therapeutic effects on acute liver injury, the mice were treated with OEA after LPS/D-Gal injection. Meanwhile, an extensively used HO-1 inhibitor, ZnPP, was administrated to mice 30 min before LPS/D-Gal stimulation to detect whether HO-1 pathway is responsible for the effects of OEA in this animal model. As shown in Figures 7A–D, OEA significantly attenuated LPS/D-Gal-induced upregulation of liver injury grade, ALT and AST levels, while ZnPP markedly blocked these protective effects of OEA in mice. In the same way, ZnPP blunted the effects of OEA on the LPS/D-Gal-induced elevation of MDA levels and the downregulation of SOD and GSH-px activities (Figures 7E–G). Collectively, these results suggested that HO-1 pathway play crucial role in the therapeutic effects of OEA on liver injury induced by LPS/D-Gal.

### OEA Alleviated H_2_O_2_-Induced Cell Injury *In Vitro*

**FIGURE 8:**
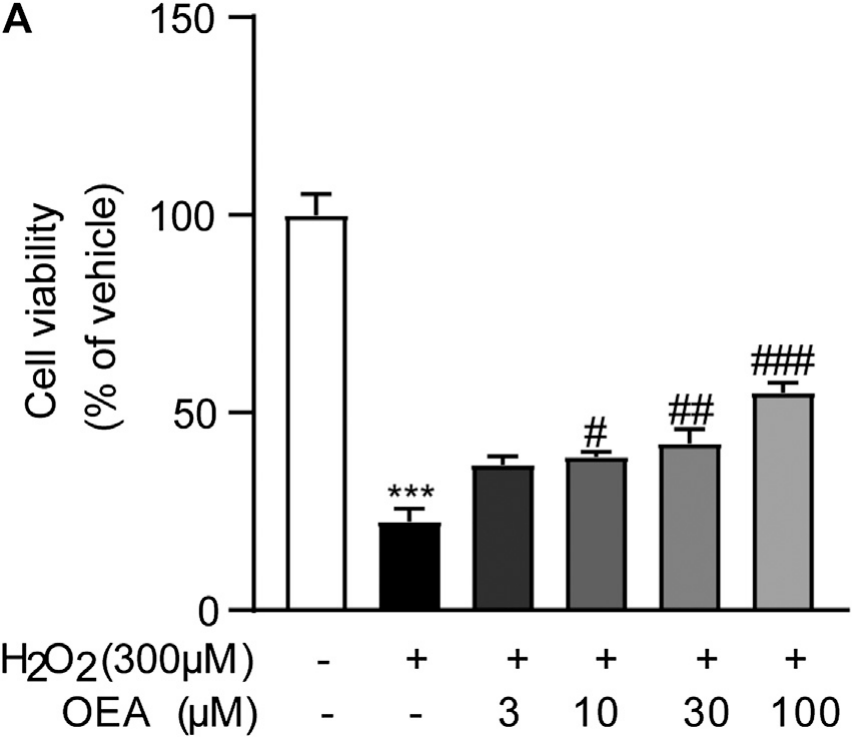
OEA attenuated H_2_O_2_-induced cytotoxicity in hepatocytes. **(A)** Effects of OEA on cell viability induced by H_2_O_2_, *n* = 5. ***p < 0.001, compared with the Control group, #*p* < 0.05, ##*p* < 0.01, ###*p* < 0.001, compared with the H_2_O_2_ group.

To evaluate whether OEA has direct impact on hepatocytes exposed to hepatotoxicant, we examined the effects of OEA on H_2_O_2_-induced cell injury in AML12 cells. As shown in Figure 8, H_2_O_2_ (300 μM) caused obvious cytotoxicity in cultured hepatocytes, while OEA (3, 10, 30, 100 μM) dose-dependently improved the decreased cell viability. These findings revealed the protective effect of OEA against hepatotoxicity *in vitro*.

### OEA Suppressed LPS-Induced Inflammation in Macrophages

**FIGURE 9:**
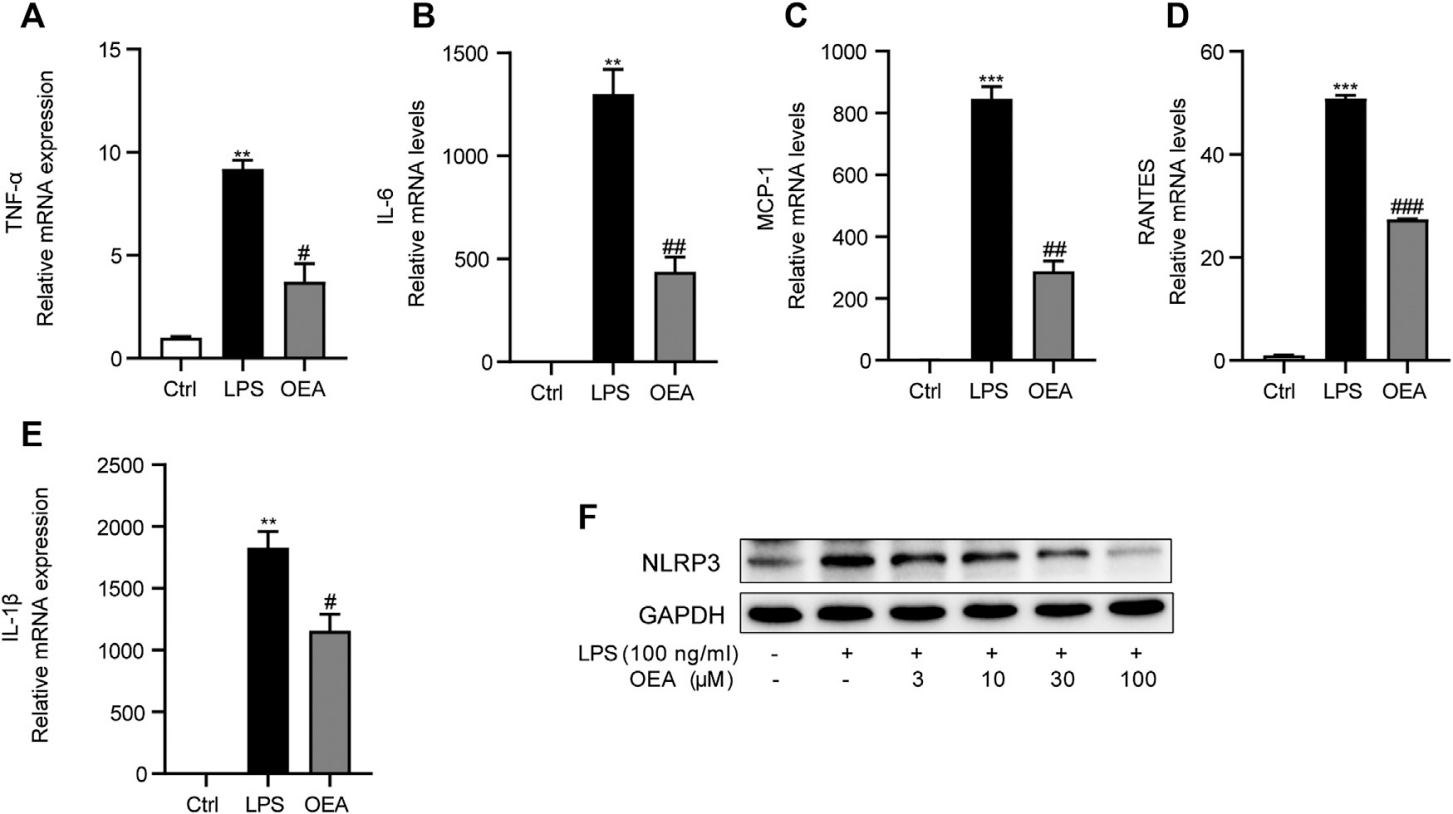
OEA inhibited LPS-induced inflammatory response in macrophages. **(A)** TNF-α, **(B)** IL-6, **(C)** MCP-1, **(D)** RANTES and **(E)** IL-1β mRNA expression in RAW264.7 macrophages treated with LPS or LPS + OEA, detected by real time PCR. **(F)** Effects of OEA (3, 10, 30, 100 μM) on the protein expression of NLRP3 induced by LPS in macrophages. Data are expressed as mean ± SEM, *n* = 3. **p < 0.01, ***p < 0.001, compared with the Ctrl group, #*p* < 0.05, ##*p* < 0.01, ###*p* < 0.001, compared with the LPS group.

To further study the effects of OEA on LPS-induced inflammatory response in macrophages, we observed the expression of inflammatory cytokines in LPS-treated macrophages in the presence or absence of OEA treatment. LPS stimulation leads to sharply elevated mRNA expression of TNF-α, IL-6, MCP1, RANTES, and IL-1β. In comparison, OEA remarkedly decreased the expression of these inflammatory factors (Figures 9A–E). Interestingly, the western blot analysis showed that OEA dose-dependently downregulated LPS-enhanced NLRP3 expression (Figure 9F). These results supported that the anti-inflammatory effects of OEA in liver may target intrahepatic macrophages.

## Discussion

Acute liver injury is a common clinical syndrome caused by multiple factors, such as viruses, drugs, alcohol, or cytokines (Patton et al., 2012; Stravitz and Lee, 2019). Without effective treatment, severe liver injury could lead to hepatocytes dysfunction or suddenly hepatic failure (Stravitz and Kramer, 2009). An increasing number of evidences demonstrates that the hepatic apoptosis, oxidative stress and inflammatory response are all associated with the development of acute liver injury, and play an important role in the pathogenetic process of disease (Li et al., 2017; Hu et al., 2020). Therefore, the agents could ameliorate apoptosis, anti-oxidant or suppress inflammation can be considered as potential therapeutic strategies for the treatment of acute liver injury.

Numerous studies have demonstrated the critical effects of OEA in multiple liver diseases (Tutunchi et al., 2020b). In our previous studies, we demonstrated that OEA possess effective pharmacological effects on diet-induced hepatic steatosis through promoting lipid β-oxidation and inhibiting lipogenesis in a high fat diet-induced NAFLD model in rats (Li et al., 2015a). We also revealed that OEA could protect against both MCD and TAA-induced liver fibrosis via PPAR-α mediated hepatic stellate cells inactivation (Chen et al., 2015). A recent randomized clinical trial revealed that OEA treatment along with calorie restriction significantly improve body composition and alleviate inflammation in obese patients with nonalcoholic fatty liver disease (NAFLD) (Tutunchi et al., 2020a). However, the functions around OEA have been found in previous reports are mainly focused on the lipid metabolism and obesity-related diseases, little is known about its action on acute liver injury. The LPS/D-Gal-induced acute liver injury model is a mature and widely used animal experimental model to mimic the clinical symptom of acute liver failure in human (Li et al., 2018; Peng et al., 2019). Therefore, we used this rodent model to assess the effects of OEA for the treatment of acute liver damages. Our present study firstly confirmed that OEA exhibited significant hepatoprotective effect against acute liver injury induced by LPS/D-Gal in mice, indicated by reducing the elevated plasma levels of ALT and AST, and significantly alleviating the histopathological changes. Moreover, our results indicated that administration of OEA could attenuate hepatic apoptosis as demonstrated by the improvement of TUNEL staining. In addition, the anti-oxidant system seemed to be firmly related to the pathological degree of acute liver injury, and the therapeutic strategies to inhibit oxidative stress could attenuate the progression of liver injury (Ni et al., 2020). We found OEA treatment could significantly suppress the expression levels of biomarkers related to oxidative stress, and enhance the activities of anti-oxidant enzymes. Besides, OEA treatment can also inhibit the expression of F4/80, which is a well-known marker of macrophage activation. Meanwhile, OEA administration could inhibit the expression of pro-inflammatory cytokines, which are key regulators of inflammation in acute liver injury. Additionally, an increasing number of researches have confirmed the crucial role of NLRP3 inflammasome signaling pathway in the development of liver injury (Al Mamun et al., 2020; Wang et al., 2020). Interestingly, our results demonstrated that OEA can markedly inhibit the activation of NLRP3 inflammasome.

Oxidative stress has been shown to be a major mechanism of acute liver injury (Cichoz-Lach and Michalak, 2014). It is reported that LPS/D-Gal could destroy the oxygen balance in the liver through induce the increase of oxidative stress-related biomarker, and decrease the anti-oxidant elements (Wei et al., 2014). OEA has been revealed that has strong anti-oxidant activities in some oxidative stress-associated diseases. For example, obese people received OEA supplementation showed improved oxidative stress in a clinical study (Payahoo et al., 2018). OEA treatment reduced the expression of oxidative genes, such as iNOS and COX-2 in atherosclerotic plaques (Fan et al., 2014). Liver oxidative stress can be assessed by detecting the concentrations of hepatic MDA, which is a naturally occurred oxidation product and one of the most commonly used biomarkers of oxidative stress in liver diseases (Galicia-Moreno et al., 2016). Anti-oxidant enzymes including SOD and GSH-px are very important antioxidant defense systems to defend against reactive oxygen species in the body. SOD could convert superoxide radicals to hydrogen peroxide, which can be further catalyzed to water and oxygen via GSH-px (Vuppalanchi et al., 2011; Arauz et al., 2016). In our study, pretreatment with OEA obviously attenuated oxidative stress as evidenced by decreased hepatic MDA levels and enhanced hepatic SOD and GSH-px activities that were altered by LPS/D-Gal injection. Nrf-2 is a key transcription factor that involved in the resistance to oxidants through regulating the expression of antioxidant and detoxication enzymes, while HO-1 is the well-established target gene of Nrf-2. It has been well documented that Nrf-2/HO-1signaling pathway play critical effects in the pathophysiology of various acute and chronic liver diseases (Xu et al., 2018; Chen et al., 2019a). For instance, Nrf-2/HO-1 pathway activation effectively alleviate liver damage during hepatic ischemia-reperfusion injury (Ge et al., 2017). Nrf-2 pathway can be a promising target for the prevention and treatment of alcoholic liver disease (Zhao et al., 2018). The chronic liver injury in nonalcoholic steatohepatitis also could be significantly attenuated via Nrf-2 signaling pathway (Chen et al., 2019b). Herein, we found that the mice receiving LPS/D-Gal have a significant decrease in protein levels of Nrf-2 and HO-1, which were significantly increased by OEA pretreatment. Moreover, the HO-1 inhibitor ZnPP abrogated the therapeutic effects of OEA on liver injury induced by LPS/D-Gal in mice, suggesting that OEA might enhance anti-oxidative defense in LPS/D-Gal treated mice through Nrf-2/HO-1 pathway.

The mechanisms responsible for acute liver injury are complex involving multiple factors. As an inflammatory disorder of the liver, the pathogenesis of acute liver injury is closely related to inflammatory response (Donnelly et al., 2016). It is well recognized that intrahepatic macrophages, the Kupffer cells, exerting crucial effects during liver homeostasis (Li et al., 2020a). Kupffer cells could be activated in response to LPS as well as other PAMPs (Peng et al., 2019). Under pathological condition, intrahepatic macrophages were activated, and numerous pro-inflammatory factors were highly expressed and secreted from activated Kupffer cells (Yang et al., 2020). Among these factors, TNF-α was generally considered as the most important cytokine leading to hepatocytes damage (Schwabe and Brenner, 2006). IL-6 is also a well-established biomarker of inflammation that significantly increased during the pathogenesis of acute liver injury. However, in contrast to TNF-α, IL-6 may have the protective effects in LPS/D-Gal-induced acute liver injury through modulating Kupffer cells polarization, which has been proved in our previous study (Li et al., 2017). Chemokines such as MCP-1 and RANTES could promote the accumulation of macrophages and the migration of leukocytes into the diseased liver, and then aggravate the inflammatory response during the development of acute liver injury (Marra and Tacke, 2014). In our present study, we found that the number of activated Kupffer cells was increased and the expression of TNF-α, IL-6, MCP1, and RANTES were all significantly up-regulated in the liver of mice treated with LPS/D-Gal. Previous studies have shown that OEA has anti-inflammatory effects in various inflammation-related disease model, including dextran sodium sulphate-induced colitis (Lama et al., 2020), high caloric diet-induced atherosclerosis (Fan et al., 2014) and LPS-induced neuroinflammation (Sayd et al., 2014). Our present study showed that the elevated number of activated Kupffer cells and increased levels of pro-inflammatory factors in LPS/D-Gal treated mice were obviously suppressed by the treatment of OEA, which indicated that OEA has potent anti-inflammatory effects in the liver of acute liver injury mice.

NLRP3 inflammasome can be activated in response to pro-inflammatory mediators such as LPS to increase the expression of cytokines such as IL-1β (Swanson et al., 2019). Accumulating evidences have revealed the critical role of NLRP3 inflammasome in the development of several liver diseases including NAFLD, liver fibrosis and acute liver injury (Al Mamun et al., 2020). Therefore, targeting the NLRP3 inflammasome has been explored as an effective strategy for the treatment of all kinds of liver diseases (Szabo and Csak, 2012). Although numerous researches have reported the protective effects of OEA on various liver diseases associated with NLRP3 inflammasome activation, whether OEA could regulate the activation of NLRP3 inflammasome has not been investigated until our present study. Kim et al. demonstrated that NLRP3 inflammasome activation is responsible for liver injury induced by LPS/D-Gal (Kim and Lee, 2013). In accordance with previous reports, we also found the elevated protein expression of IL-1β, NLRP3 and caspase-1 in the liver of LPS/D-Gal-challenged mice, which were significantly alleviated by OEA treatment. These results in our study indicated that OEA could attenuate the enhanced NLRP3 activation in acute liver injury induced by LPS/D-Gal. As an endogenous cytoprotective enzyme, HO-1 was proved has the ability to suppress the NLRP3 signaling pathway and then inhibit LPS/D-Gal-induced hepatic inflammation in mice (Kim and Lee, 2013). Interestingly, our present study indicated the anti-oxidant role of OEA via up-regulate Nrf-2 and HO-1 expression, we assume that HO-1 might also mediated the inhibitory effect of OEA on NLRP3 inflammasome activation, which needed further study to deeply investigate it.

The nuclear receptor PPAR-α belongs to the PPARs superfamily, which comprising PPAR-α, PPAR-γ, and PPAR-β/δ. According to previous reports, PPAR-α is mainly expressed in liver, heart and muscle, as well as other metabolically active tissues, and play crucial effects in modulating lipid metabolism and inflammation (Abbott, 2009). Several studies have revealed that PPAR-α is associated with the development of multiple liver diseases. The expression of PPAR-α in liver is negatively correlated with the severity of liver injury in patients with NAFLD (Francque et al., 2015). Jiao et al. have demonstrated that the hepatic expression of PPAR-α in LPS/D-Gal-induced acute liver injury was markedly reduced, which phenomenon was also observed in our present study (Jiao et al., 2014). As a high affinity endogenous ligand of PPAR-α, a variety of pharmacological activities of OEA were mediated by the PPAR-α signaling pathway. Fu et al. revealed that OEA regulates body weight through PPAR-α mediated reduction of food intake (Fu et al., 2003). Our previous study demonstrated that OEA promotes fatty acid β-oxidation through initiate the transcription of PPAR-α in diet-induced NAFLD in rats (Li et al., 2015a). In addition, OEA could attenuate liver fibrosis in wild type mice, but not in PPAR-α knockout mice (Chen et al., 2015). The results of this study showed that the reduced expression of PPAR-αwas significantly increased in LPS/D-Gal-treated mice received OEA treatment, indicating that the protective effects of OEA against acute liver injury may be dependent on the upregulation of PPAR-α.

In summary, our current study demonstrated the protective effects of OEA on LPD/D-Gal-induced acute liver injury in mice. What’s more, we provided a deep understanding of the molecular mechanisms under these effects, which may be due to its anti-inflammatory and anti-oxidant activities through increasing PPAR-α expression levels and modulating Nrf-2/HO-1 and NLRP3 inflammasome signaling pathways. The present study suggests that OEA administration may be a potential new strategy for clinical treatment of acute liver diseases.

## Data Availability Statement

The original contributions presented in the study are included in the article/Supplemental Material, further inquiries can be directed to the corresponding authors.

## Ethics Statement

The animal study was reviewed and approved by Experimental Animal Ethical Committee of Ningbo University.

## Author Contributions

JH, LL, and YZ designed the experiments. JH, ZZ, HY, and JY performed experiments. JH, HM, and LL analyzed the data. YZ and HM contributed reagents and materials. JH and LL wrote the manuscript. All authors approved the final version of the manuscript.

## Funding

This research was funded by the National Natural Science Foundation of China (No. 91856126, No. 81870606), the Natural Science Foundation of Zhejiang Province (No. LQ21H030002) and the Ningbo Natural Science Foundation (No. 2019A610209).

## Conflict of Interest

The authors declare that the research was conducted in the absence of any commercial or financial relationships that could be construed as a potential conflict of interest.
