# Supplementary figures and images for: Oleoylethanolamide Protects Against Acute Liver Injury by Regulating Nrf-2/HO-1 and NLRP3 Pathways in Mice
Source: Front Pharmacol. 2021 Jan 18;11:605065. doi: 10.3389/fphar.2020.605065 (PMC7848133; doi:10.3389/fphar.2020.605065)

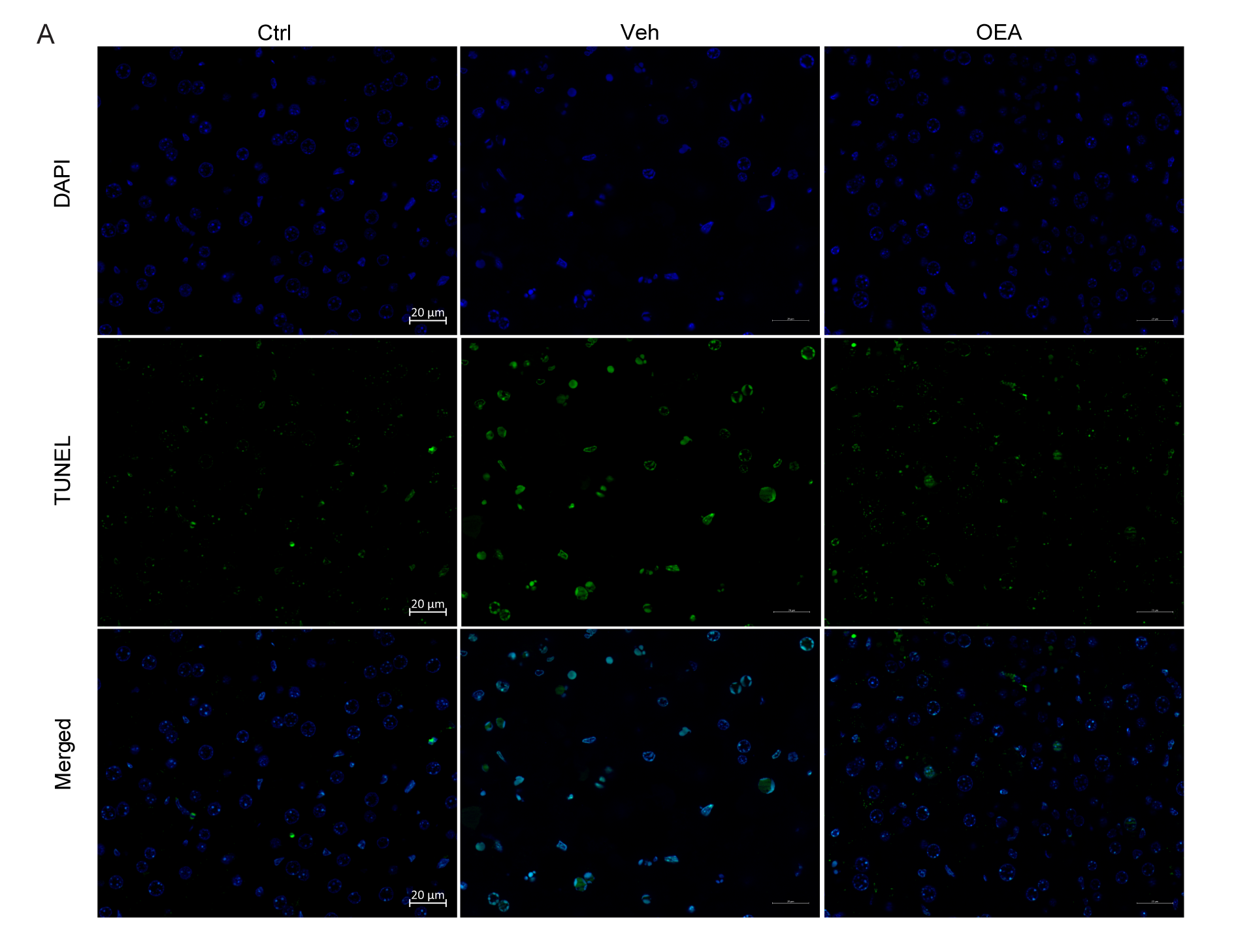

Supplement: Supplementary file 1 [file image1.tif]
